# Hierarchically structured nanoporous carbon tubes for high pressure carbon dioxide adsorption

**DOI:** 10.3762/bjnano.8.115

**Published:** 2017-05-24

**Authors:** Julia Patzsch, Deepu J Babu, Jörg J Schneider

**Affiliations:** 1 Fachbereich Chemie, Eduard-Zintl-Institut für Anorganische und Physikalische Chemie, Alarich-Weiss-Strasse 12, Technische Universität Darmstadt, 64287 Darmstadt, Germany

**Keywords:** carbon dioxide adsorption, carbon tubes, gas adsorption, mesoporous carbon

## Abstract

Mesoscopic, nanoporous carbon tubes were synthesized by a combination of the Stoeber process and the use of electrospun macrosized polystyrene fibres as structure directing templates. The obtained carbon tubes have a macroporous nature characterized by a thick wall structure and a high specific surface area of approximately 500 m²/g resulting from their micro- and mesopores. The micropore regime of the carbon tubes is composed of turbostratic graphitic areas observed in the microstructure. The employed templating process was also used for the synthesis of silicon carbide tubes. The characterization of all porous materials was performed by nitrogen adsorption at 77 K, Raman spectroscopy, infrared spectroscopy, thermal gravimetric analysis (TGA), scanning electron microscopy (SEM) as well as transmission electron microscopy (TEM). The adsorption of carbon dioxide on the carbon tubes at 25 °C at pressures of up to 30 bar was studied using a volumetric method. At 26 bar, an adsorption capacity of 4.9 mmol/g was observed. This is comparable to the adsorption capacity of molecular sieves and vertically aligned carbon nanotubes. The high pressure adsorption process of CO_2_ was found to irreversibly change the microporous structure of the carbon tubes.

## Introduction

Nanostructured carbon and silicon carbide materials have numerous potential applications. Structured carbons such as graphene, carbon nanotubes, carbon fibres or hierarchical porous carbons were successfully tested as potential material for catalysis [1], gas sensors [2], electronic devices [3] and for gas adsorption [4]. Activated carbons (ACs) are widely used for gas adsorption because of their straightforward production, low cost and thermal stability [5–7]. Nevertheless, the excellent adsorption characteristics of ACs are often outweighed by their irregular and undefined pore structure. As a consequence, the gas adsorption process is complex and a multistep regeneration process is needed to complete the outgassing of the adsorbed gases. To counter this problem, two fundamental methods are used for increasing their adsorption capacity: (i) morphological structuring to increase the surface area and (ii) modification of the tube surface with functional groups to enhance the adsorbent/adsorbate attraction. For (i), new carbon materials have been studied as adsorbents such as ordered mesoporous carbon [8], single-wall carbon nanotubes (CNTs) [9], multiwall CNTs [10], double-wall aligned CNTs [11] as well as graphene [12]. In the case of (ii), oxygen groups such as C–O and C=O were introduced on the carbon surface to enhance the adsorption of gases such as CO_2_ [11].

Silicon carbide is attractive as a potential material for catalysis and electronic and photonic devices due to its semiconducting nature with a wide band gap, excellent mechanical properties, chemical inertness and thermal conductivity [13–17]. Especially, one-dimensional SiC in the form of nanowires or nanorods show outstanding elasticity and mechanical strength. A Young’s modulus of 600 GPa was measured for SiC wires [18–19]. Different templating methods were used for structuring such as the two-step synthesis using preceramic polymers as precursors (e.g., polycarbosilanes) [13,20–21], carbo-thermal reduction at high temperatures (≈1300 °C) [22–23] or magnesio-thermic reduction at moderate temperatures (≈700 °C) [24]. Following these approaches, SiC nanotubes were successfully synthesized by reaction with CNTs [25–26], with porous aerogels [27], fibres [28], and ordered mesoporous SiC structures created by nanocasting [29]. All of these approaches have allowed for the synthesis of ordered hierarchical macro–mesoporous materials [24]. Electrospinning is a versatile technique for the synthesis of different one-dimensional forms such as fibers, tubes or wires for various applications such as gas sensors [30–32] or photoelectrodes for dye-sensitive solar cells [33]. This technique has also be extended for the synthesis of one-dimensional metal oxide nanomaterials [34–37].

Herein, we introduce a process which allows highly porous carbon tubes as well as nanocrystalline silicon carbide tubes to be obtained. To obtain these materials, a polymer was employed and used as the carbon source and template to mold a spherical structure of silica particles obtained by the Stoeber process. After a carbonization step, a second thermal treatment was employed to obtain either SiC tubes or a selective hydrofluoric acid (HF) etching was used, which leads to pure carbon tubes. Due to their high surface area and porous nature, the carbon tubes are an interesting material for gas storage applications. Consequently, high pressure gas adsorption studies of carbon dioxide were carried out on this material.

## Experimental

### Materials

Polystyrene (PS, pro-plast from BASF), tetraethylorthosilicate (TEOS, ABCR), ammonia (NH_3_, Grüssing), dimethylformamide (DMF, Merck), tetrahydrofuran (THF, Merck), and ethanol (EtOH, Brenntag) were used as received without further purification.

#### Synthesis of polystyrene fibres (**1**)

In a similar manner as described in [38], electrospinning was performed in a homebuilt apparatus. Polystyrene (PS) fibres were electrospun from a 16 wt % PS THF/DMF 3:2 solution. After aging the spinning solution overnight, it was loaded into a glass syringe equipped with a stainless-steel needle (0.8 × 20 mm). The voltage applied to the needle tip was kept at 30 kV and the distance between the copper counter electrode and the tip was 16 cm. The spun fibres were dried at room temperature overnight. A plasma treatment process was used for functionalizing the PS fibre surface. The plasma functionalization was carried out on a radio frequency (13.56 MHz) parallel plate plasma setup (Femto, Diener electronic GmbH, Germany) with a maximum power rating of 300 W. After the chamber was evacuated to low-pressure residual air (0.3 mbar), the PS fibre samples (**1**) were treated with an oxygen plasma generated at 20 W for one minute.

#### Synthesis of silica@polystyrene composite tubes (**2**) and silica@carbon composite tubes (**3**)

Analogous to our previous work [38], a modified Stoeber method was used to coat the plasma-treated, and thus oxo-functionalized, PS fibres with a silica shell. In a typical reaction, the oxo-functionalized PS fibres were suspended in EtOH and a TEOS/H_2_O/NH_3_ mixture (molar ratio of 1:4.4:24.9) was added under stirring. After 18 h, the fibres were filtered and rinsed with ethanol and dried at 80 °C overnight. Additionally, a sol suspension of TEOS/ethanol/H_2_O/HCl (molar ratio 1:124:8.1:0.5) was sprayed onto the PS fibres with a commercial air brush gun. The obtained silica@polystyrene composite (**2**) was treated at 250 °C for 16 h under air and at 950 °C for 4 h under nitrogen atmosphere, yielding the silica@carbon composite tubes (**3**).

#### Synthesis of carbon tubes (**4**)

An HF solution was used to remove the silica shell of the silica@carbon composite (**3**). The as-obtained carbon tubes were washed with water and dried at 80 °C. The obtained carbon tubes (**4**) were finally treated at 1300 °C or 1600 °C for 1 h.

#### Synthesis of silicon carbide tubes (**5**)

The silica@carbon composite (**3**) sample was heated up to 1600 °C for 1 h under vacuum. The resulting material was treated with HF solution, followed by calcination at 750 °C for 4 h under air and etched with HF solution a second time to obtain the pure silicon carbide (SiC) tubes (**5**). The SiC tubes were finally washed with water and dried at 80 °C after the etching steps.

#### Physical characterisation methods

Nitrogen adsorption–desorption isotherms were measured at 77 K with a Nova 3000e (QuantaChrome) instrument after sample pretreatment at 250 °C for 18 h. The specific surface area was calculated by the Brunauer–Emmett–Teller (BET) equation from a linearized isotherm equation between *P*/*P*_0_ 0.035 and 0.2 and the pore size was calculated by density functional theory (DFT) for slit pores. The scanning electron microscopy (SEM) micrographs were obtained using a Philips XL 30 FEG (20 kV) instrument equipped with an EDX (energy dispersive X-ray) detector using an aluminum sample holder. High-resolution transmission microscopy (HRTEM) analysis was performed on lacey carbon copper grids (300 mesh) at a G2F20 (Tecnai) at the ERC-Jülich in Germany. IR measurements were performed on a Nicolet 6700 spectrometer with an ATR Smart Performer unit from Thermo Fisher. Raman spectroscopy was carried out using a LabRAM high-resolution microscope (Horiba Jobin Yvon, model HR 800). The excitation source was a 514.5 nm Ar laser. High pressure CO_2_ adsorption measurements were carried out in a self-built volumetric setup equipped with three pressure transducers in the range 0–3 bar, 0–30 bar and 0–100 bar with an accuracy of 0.05% of the maximum pressure rating. The all stainless steel construction was made from Swagelok^®^ tubes and fittings. A water bath was used for maintaining isothermal conditions and K-type thermocouples monitored the gas temperature in the storage vessel as well as in the adsorption chamber. The setup was calibrated at 25 °C using high purity N_2_ (99.999%) and the density values were obtained from the NIST database. The calibration values were validated by measuring CO_2_ (99.998%) adsorption on a Norit R1 extra™ device at 25 °C following the procedure of Möllmer et al. [39]. The values were found to be in good agreement with those reported in literature for a pressure range of 0–40 bar [40]. In a typical measurement, about 80 mg of the sample was weighed accurately after degassing at 150 °C and loaded in to the adsorption chamber. The sample was further subjected to an in situ activation process by overnight heating in vacuum at a temperature of 150 °C. CO_2_ adsorption measurements were carried out at 25 °C and CO_2_ density values, for a given pressure and temperature, were obtained from the NIST database. As the errors tend to accumulate in the volumetric measurement, only three measurements were made in a single cycle and the sample was then subjected to reactivation at 150 °C in vacuum. Since the specific volume as determined from the He measurement was less than the uncertainty of the measurement, adsorption is expressed in reduced mass (Ω) given by: 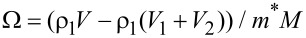
mmol/g, where ρ_1_ and ρ_2_ are the bulk density of CO_2_ before and after expansion, respectively, in kg/m^3^. *V*_1_ and *V*_2_ represent the volume of the gas storage chamber and adsorption chamber, respectively, in cm^3^, m* is the sample mass expressed in g and *M* is the molar mass of CO_2_ expressed in g/mol.

## Results and Discussion

**Figure 1:**
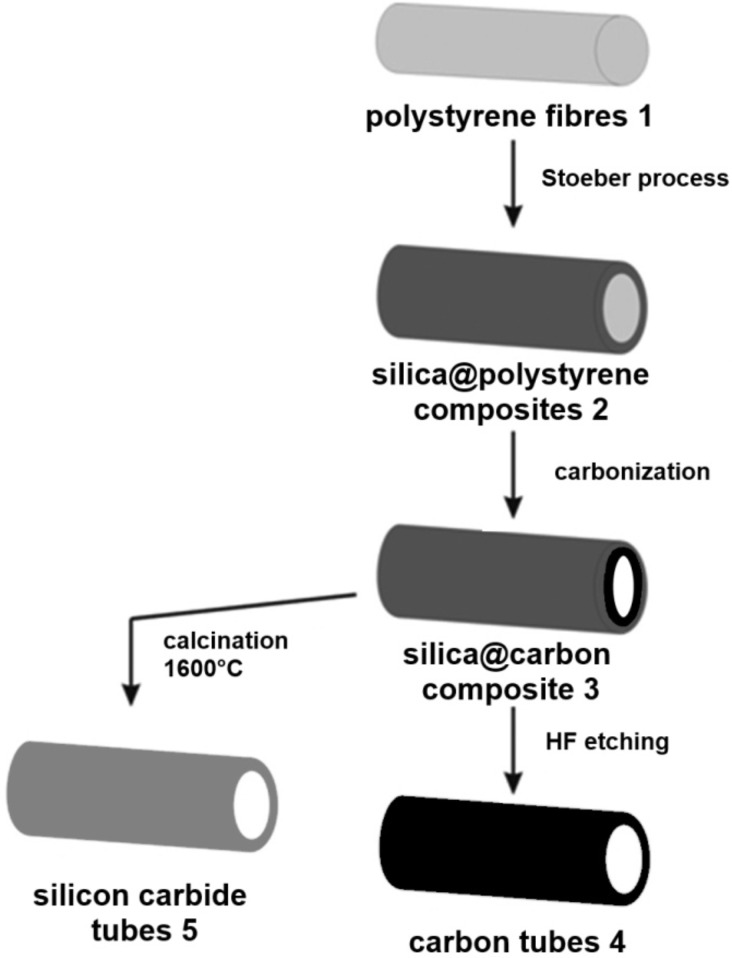
Schematic drawing showing the formation pathways leading to carbon tubes (4) and silicon carbide tubes (5) starting from electrospun polystyrene fibres (1).

In Figure 1 the overall synthesis strategy for the preparation of carbon tubes (**4**) and SiC tubes (**5**) is shown schematically. PS fibres (**1**) prepared by the electrospinning technique were subjected to a plasma treatment for effective tethering of the silica precursors. The silica@polystyrene green body composite (**2**) is obtained by the addition of silica particles [38] via the Stoeber process. The sample is subsequently heat treated to obtain the silica@carbon composite (**3**). Composite **3** is the starting composition and morphology for the synthesis of the carbon tubes **4** and the silicon carbide tubes **5** which both require the removal of the silica shell.

**Figure 2:**
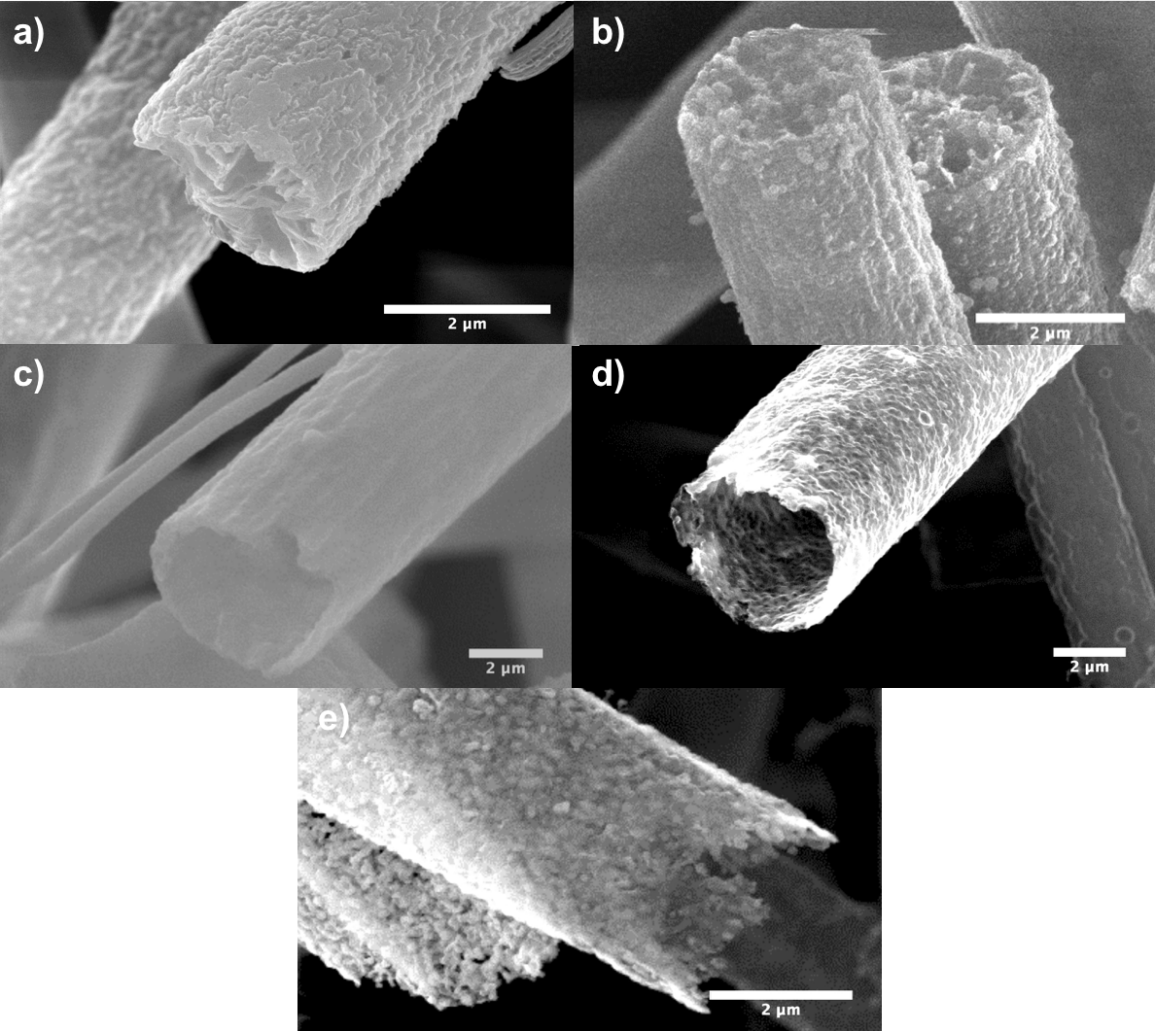
SEM images of (a) polystyrene fibres (**1**), (b) silica@polystyrene composite fibres (**2**), (c) silica@carbon composite fibres (**3**), (d) carbon tubes (**4**) and (e) silicon carbide tubes (**5**).

The electrospun PS fibres have an average diameter of about 2.5 µm as revealed by the SEM images (Figure 2a). A homogeneous coverage of silica spheres of 200 nm diameter obtained from the Stoeber process is observed on the fibre surface of **1** (Figure 2b) [38]. After heat treatment, a silica@carbon material **3** with a hollow tube structure results (Figure 2c). During the processing at 250 °C under air, the PS melts and surrounds the silica spheres. During the final carbonization step, this polymer layer is transformed into carbon. If the silica shell is removed by etching, self-supporting carbon tubes **4** remain (Figure 2d).

Due to the carbonization of the molten PS, the remaining carbon forms an interconnected porous carbon framework structure which allows the infiltration of the etching solution. Silicon carbide tubes (**5**) (Figure 2e) with a wall thickness of 140 nm were obtained after heating the silica@carbon tubes (**3**) under vacuum at 1600 °C followed by a further purification step of the SiC/C/SiO_2_ intermediate. During the conversion process, silica and carbon react according to SiO_2_ + C → SiC + CO_2_. The formed carbon layer serves as the template *and* carbon source from which the silicon carbide is formed. It furthermore prevents particle agglomeration and reduces the loss of unstable SiO species which are formed during the conversion reaction. The conversion process thus represents a micro-adaption of the well-known Acheson process for SiC formation. As a result of the conversion process, the shell of **5** is composed of a layer of interconnected SiC particles which are formed from the monolayer of SiO_2_ spheres on the PS fibres. The latter served as the templating structure as described previously by us [38].

### Characterization of carbon tubes (**4**)

**Figure 3:**
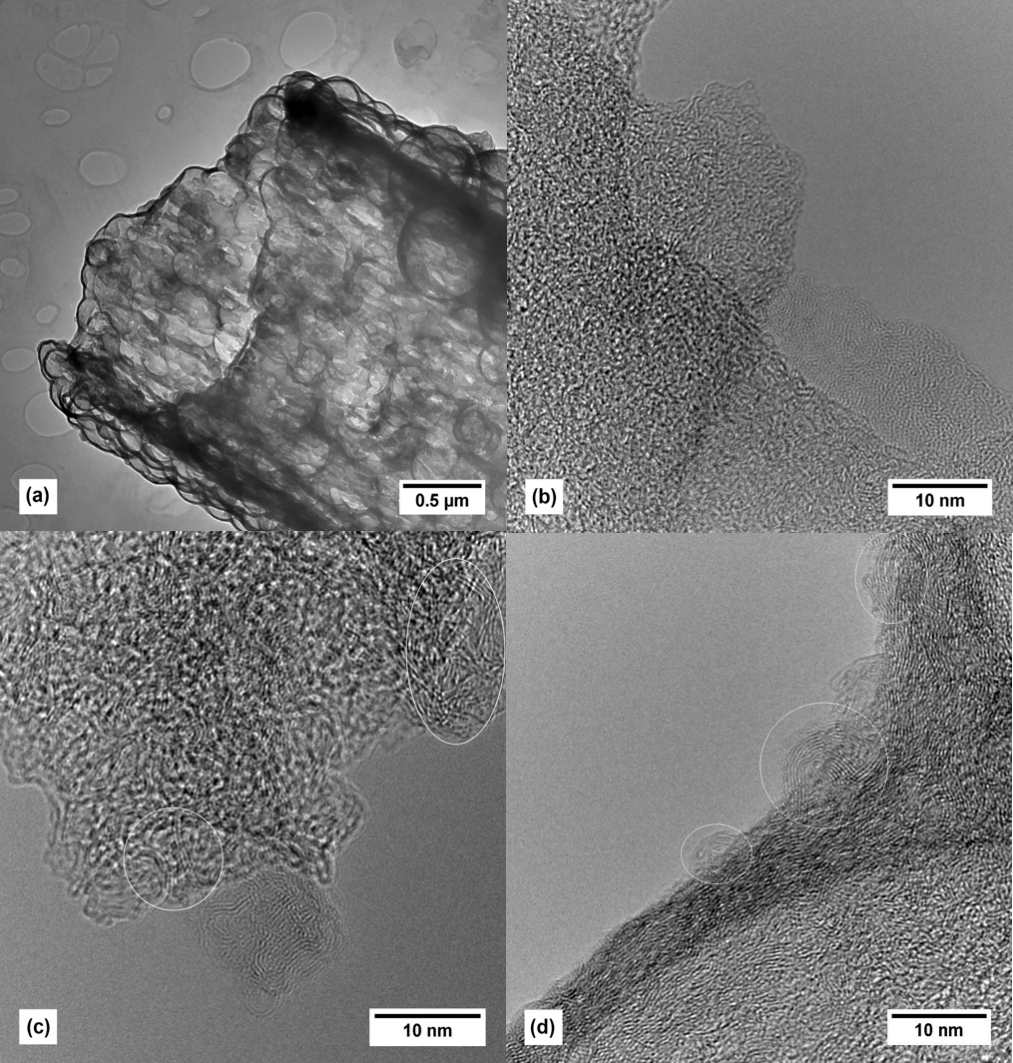
TEM images of the carbon tubes (**4**) calcined at 950 °C (a,b), 1300 °C (c) and 1600 °C (d). Circles indicate graphitic regions with fullerene- or onion-like structural moieties.

Figure 3a shows TEM images of the carbon tubes (**4**). The tube walls are composed of rounded hollow particles which form an interconnected system of irregular macropores. This is due to the templating effect of the molten PS which encloses the Stoeber silica particles. The carbonization process generates graphitic regions even at 950 °C (Figure 3b). Their crystallinity increases by further increasing the carbonization temperature up to 1300 °C and 1600 °C, giving rise to the formation of graphitic regions with turbostratic ordering of the graphitic areas (Figure 3c,d). These are typical for glassy carbon related materials as well as carbon structures containing fullerene- or carbon-onion-like fragments [41–44].

**Figure 4:**
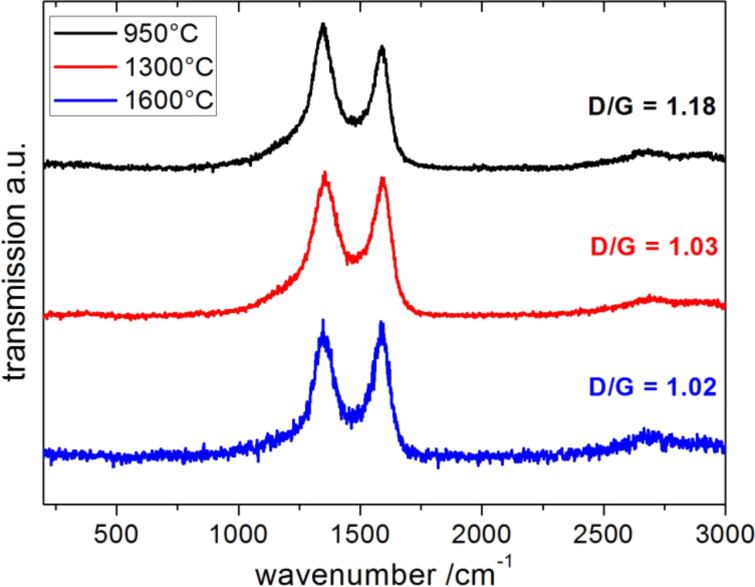
Raman spectra of the carbon tubes (**4**) carbonized at 950 °C (black/top), 1300 °C (red/middle) and 1600 °C (blue/bottom).

Figure 4 shows the Raman spectra of the carbon tubes (**4**) carbonized at different temperatures. The D-band at 1345 cm^−1^ is characteristic for sp³ carbon and the G-band at 1588 cm^−1^ for sp² carbon. The D/G ratio is characteristic for a low temperature glassy carbon type material [41]. Consistent with the TEM results, the G-band in the Raman spectrum (D/G = 1.18, 1.03, 1.02 for 950 °C, 1300 °C, 1600 °C, respectively) confirms the presence of graphitic carbon. Furthermore, the decrease in the D/G ratio observed with an increase in temperature corroborates the similar trend of the increased graphitic ratio observed from TEM measurements.

**Figure 5:**
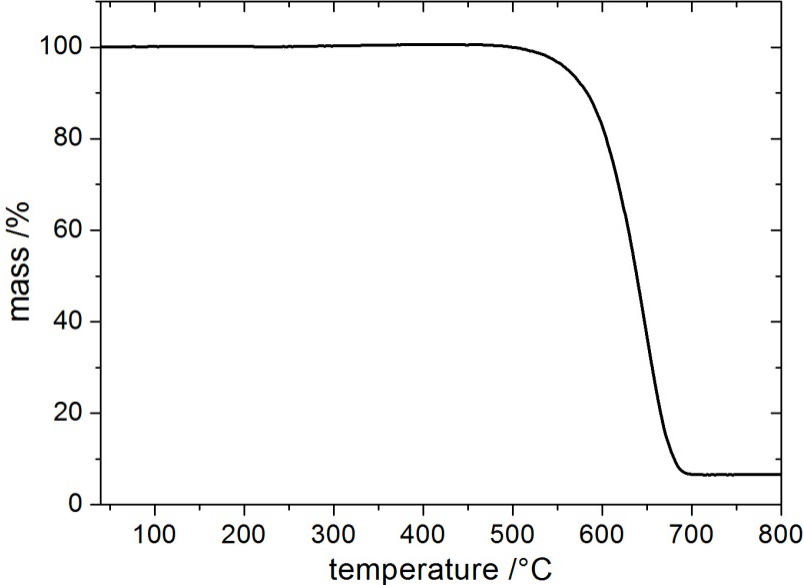
Thermogravimetric plot of the decomposition of the carbon tubes (**4**) carbonized at 950 °C in air.

The thermal decomposition behavior of the carbon tubes (**4**) carbonized at 950 °C was examined under oxygen atmosphere (Figure 5) using thermal gravimetric analysis. The decomposition starts at about 530 °C indicating a high thermal stability of the carbon material. The carbon tubes (**4**) do not decompose completely even at temperatures of 800 °C. A ceramic residue (6.5%) remains as a white powder which was identified as SiO_2_ by EDX analysis. Obviously, a small amount of the oxide particles is completely embedded in the molten PS fibres during the thermal treatment so that a complete etching by HF is not possible.

**Figure 6:**
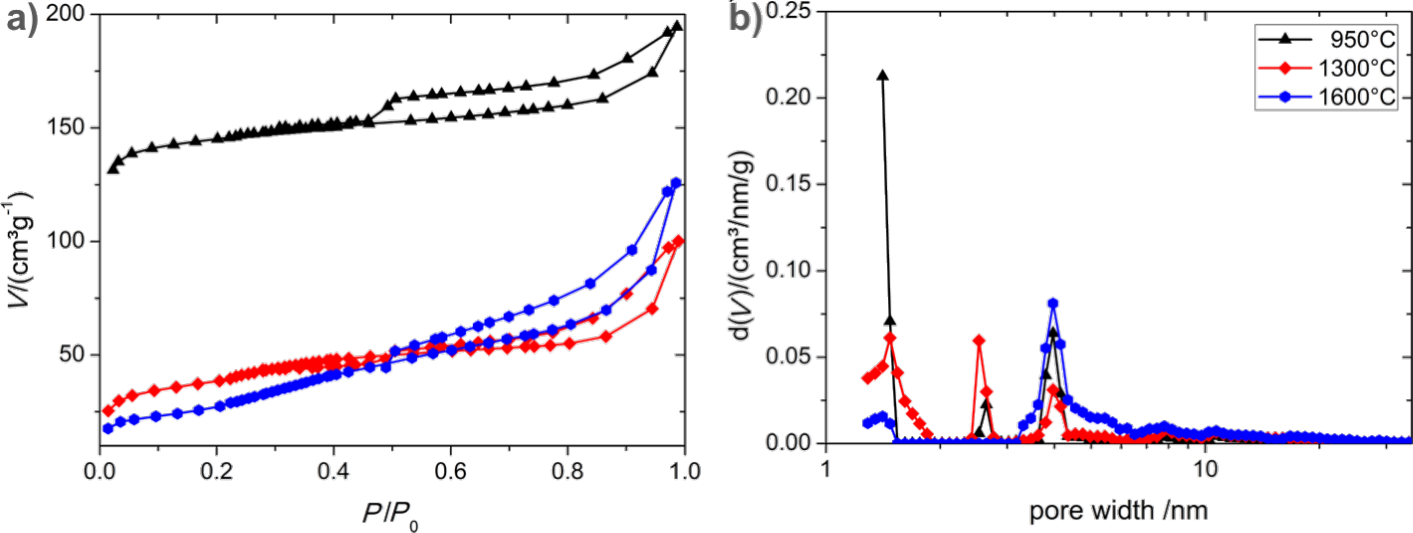
(a) Nitrogen adsorption–desorption isotherms at 77 K and (b) pore size distribution function from adsorption (DFT, slit pore model) for carbon tubes (**4**) which are carbonized at 950 °C (black/triangles), 1300 °C (red/diamonds) and 1600 °C (blue/circles).

With the observed capillary condensation at high relative pressure, the adsorption isotherm of carbon tubes (**4**) (Figure 6a) resembles a type-IVa isotherm [45], however, with a high microporous content. These micropores are responsible for the observed steep increase in the adsorption at very low relative pressure (Figure 6b). The pore width distribution calculated by the DFT method (Table 1) shows mesopores in the range of 2.6 nm and 4.0 nm in addition to the micropores of 1.4 nm. The comparison of the BET calculation for the whole surface and the *t*-plot method for the micropores, reveals that just 22% of the pore volume and 6% of the surface are generated by pores in the mesopore regime. The BET surface decreases drastically with the increasing carbonization temperature due to a lower amount of micropores. Meanwhile the mesopore content increases. This observation corresponds well with commercial glassy carbon materials which are processed at low temperature and obey a narrow pore width distribution around 1 nm [46] in comparison to high temperature glassy carbons with mesopores in the range of 5 nm [41]. This difference of the two materials processed under different temperature conditions might be due an increase of graphitic onion-like substructures which form under the high temperature treatment from lesser graphitic-like material. The growth of these sp² zones leads to a decrease of open adsorption sites and leads to an obstruction of former micropores.

**Table 1:** BET surface area, micropore surface area calculated by *t*-plot, average pore diameter calculated by DFT and *t*-plot for carbon tubes **4**.

Temperature (°C)	*A*_BET_ (m²/g)	*A*_t-Plot_ (m²/g)	*V*_DFT_ (cm³/g)	(*V*_t-Plot_) cm³/g

950	540	510	0.27	0.21
1300	135	104	0.14	0.06
1600	120	10	0.18	0
950 (after CO_2_ treatment)	286	179	0.26	0.08

### Characterization of silicon carbide tubes (**5**)

**Figure 7:**
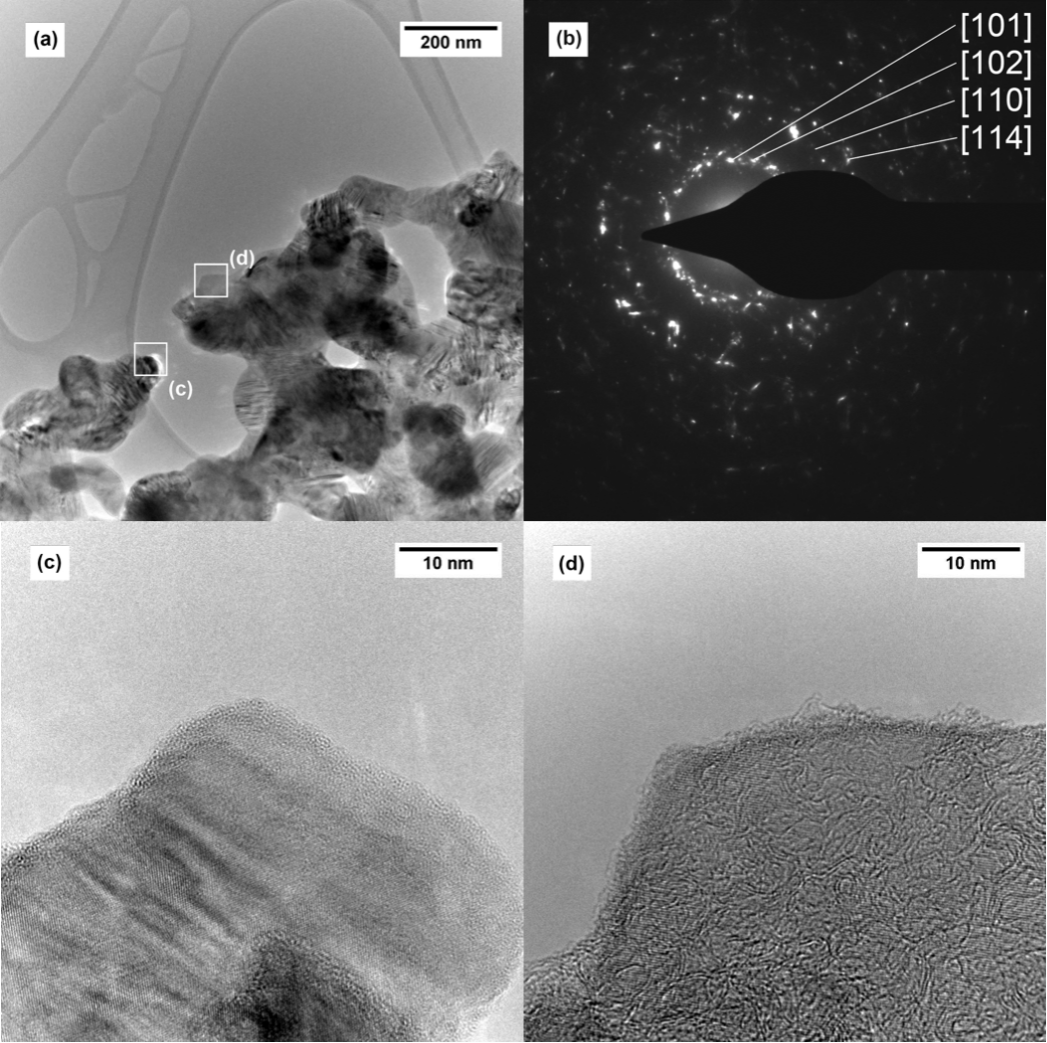
TEM images of a SiC tube wall with interconnected, crystalline SiC particles (a) and the corresponding SAED pattern (b). Individual SiC crystallites are shown in (c) and the SiC surface with a layer of microcrystalline carbon on the SiC surface is shown in (d).

Figure 7 shows TEM images of the silicon carbide tubes (**5**) and the corresponding SAED pattern. All reflexes correspond to the <101>, <102>, <110> and <114> reflexes of the Moissanite modification (JCPDS-Nr. 22-1127, 4H) of SiC. Additionally, spurious residues of microcrystalline carbon can be observed even after calcination temperatures of 750 °C under air.

**Figure 8:**
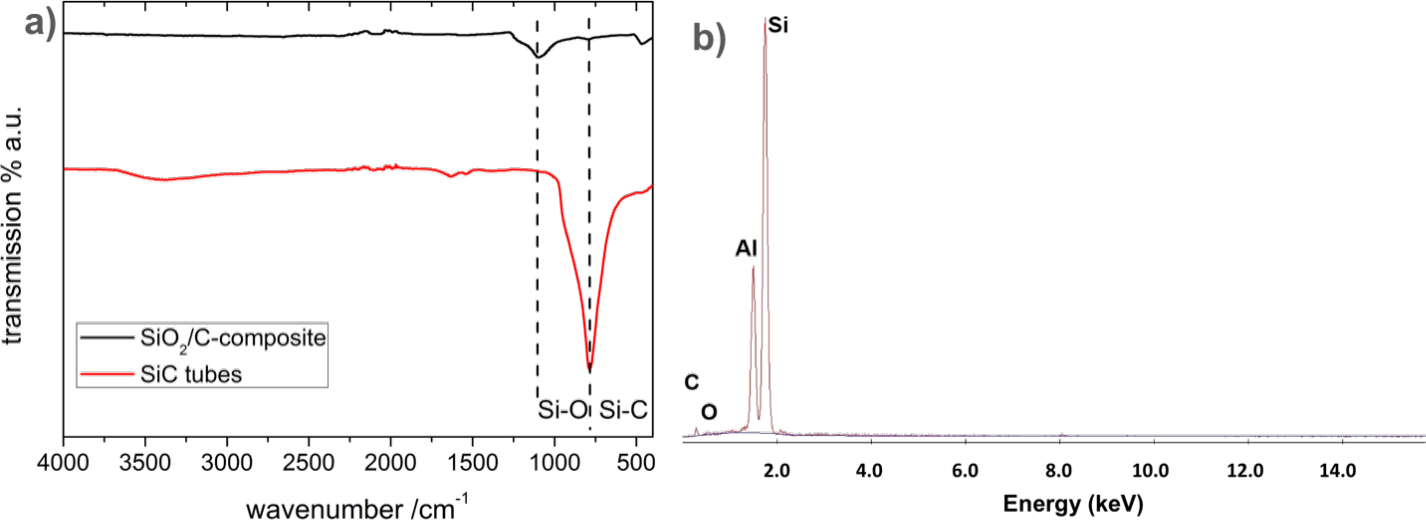
(a) IR spectra of the silica@carbon composite (**3**) (black/top) and the silicon carbide tubes (**5**) (red/bottom). (b) EDX spectra of the SiC tubes (**5**) revealing 29.6 wt % C; 5.22 wt % O; 65.2 wt % Si; 46.7 atom % C; 6.42 atom % O; 46.0 atom % Si.

The IR spectrum of the silica@carbon composite (**3**) compared to the SiC tubes (**5**) obtained after thermal treatment and purification shows a distinct change (Figure 8a). The Si–O vibration for **3** at ν = 1060 cm^−1^ disappears and the Si–C valence vibration of **5** at ν = 782 cm^−1^ appears. Figure 8b shows the EDX spectra of **5** with a Si/C ratio close to 1:1. About 6.4 atom % of oxygen is also observed and can be attributed to a residue of SiO_2_ within the SiC matrix which cannot be removed by HF etching. This observation corroborates with that observed in the formation of the carbon tubes (**4**).

### High pressure carbon dioxide adsorption on carbon tubes (**4**)

**Figure 9:**
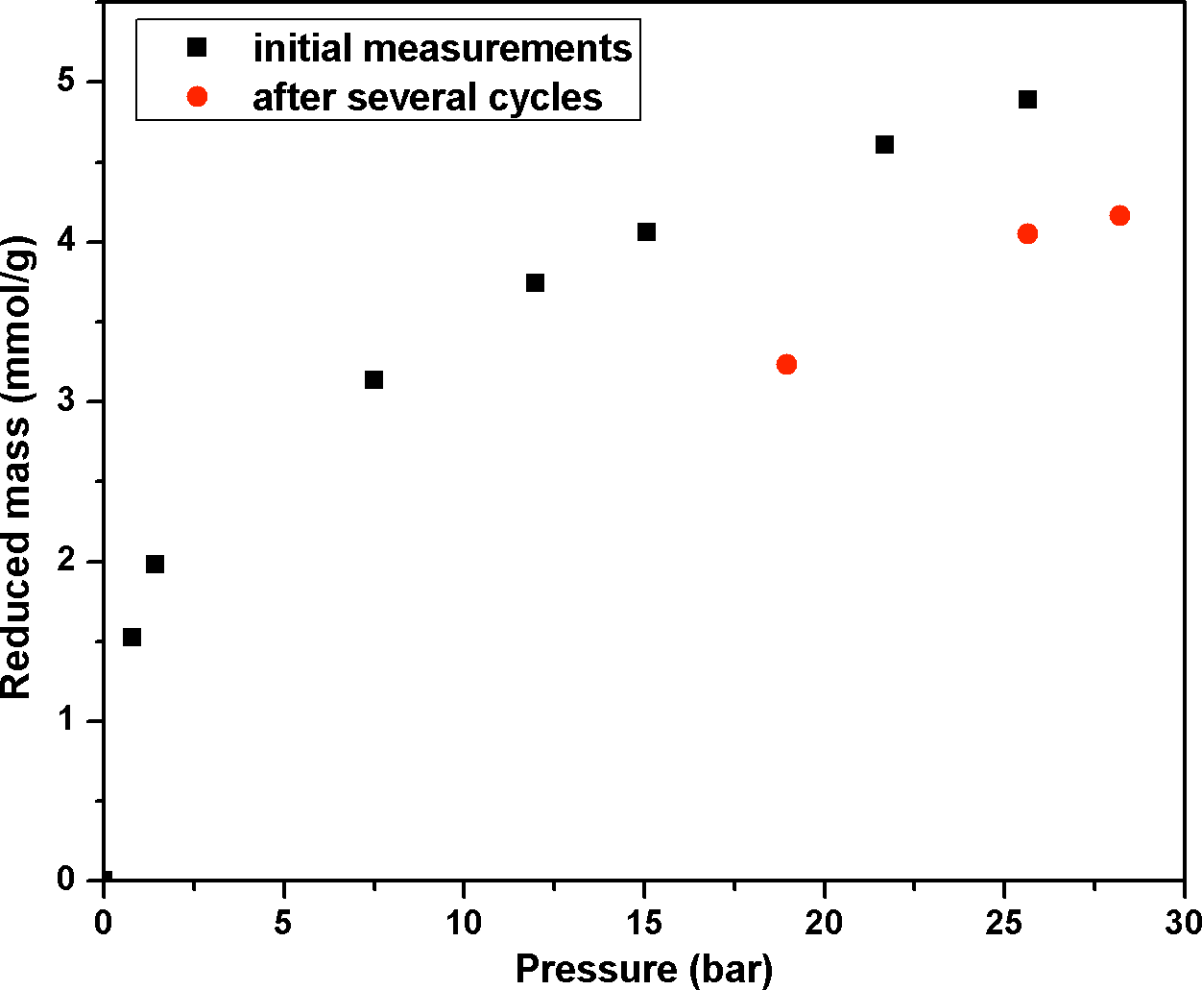
High pressure carbon dioxide adsorption isotherm at 25 °C for carbon tubes (**4**) carbonized at 950°C.

**Figure 10:**
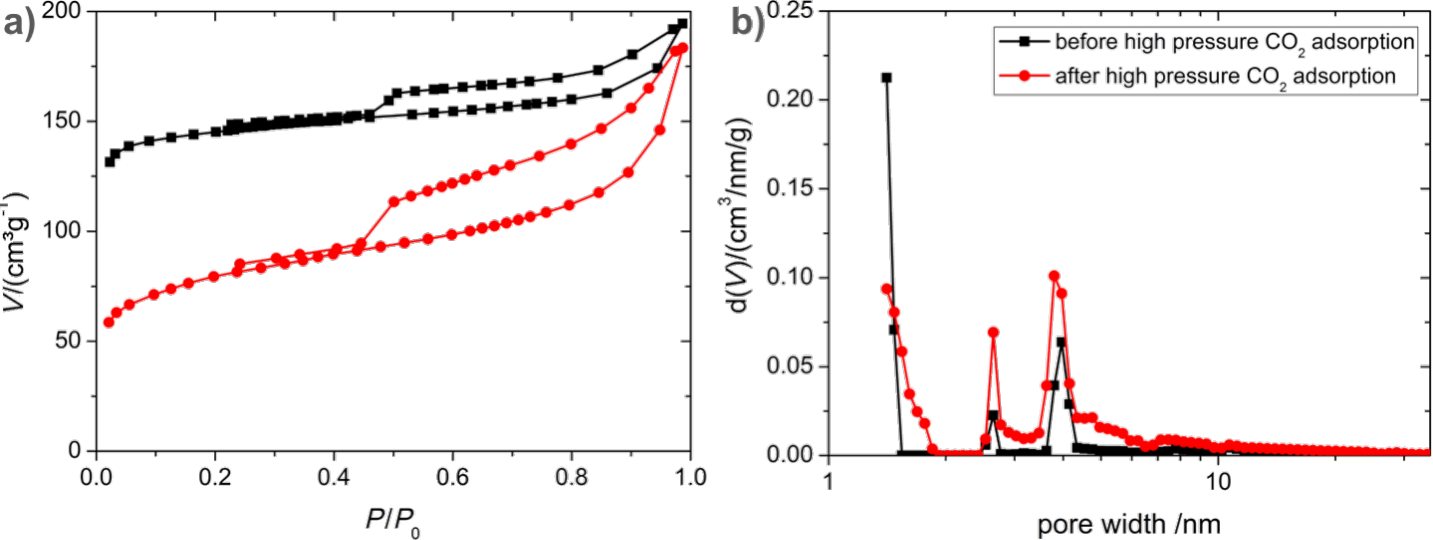
Nitrogen adsorption–desorption isotherms at 77 K (a) and pore size distribution from adsorption (DFT, slit pore model) (b) for carbon tubes (**4**) before (black/squares) and after (red/circles) CO_2_ adsorption.

Figure 9 illustrates the high pressure adsorption of carbon dioxide on the carbon tubes (**4**) carbonized at 950 °C. Due to the high microporous content, a considerable adsorption of CO_2_ is observed even at atmospheric pressure. The amount of CO_2_ adsorbed increases with an increase in the CO_2_ pressure. At 26 bar, an adsorption capacity of 4.9 mmol/g is observed. This is comparable to the adsorption capacity of molecular sieves [47] and vertically aligned carbon nanotubes [11]. However, after about ten adsorption and regeneration cycles, the adsorption capacity decreases to 4.1 mmol/g. It is interesting to note that the decrease in adsorption capacity was observed only at high pressure (>15 bar), while at low pressure, the adsorption capacity remains the same. Cazorla-Amorós et al. [48] have shown that at 298 K and near ambient pressure, CO_2_ molecules adsorb on the ultramicropores (<0.7 nm), while at high pressure, CO_2_ adsorption occurs on the supermicropores of the adsorbent. Since almost no change is observed in the adsorption isotherm at low pressure, and the decrease in adsorption is only observed at high pressure for **4**, it is believed that the amount of supermicropores (0.7 nm to 2 nm) present in the sample has decreased. This is indeed confirmed by the N_2_ adsorption measurements at 77 K on the carbon tubes (**4**) subjected to several cycles of high pressure CO_2_ adsorption (Figure 10a). A considerable reduction in the BET specific surface area from 540 m^2^/g to 280 m^2^/g is observed after several cycles of adsorption. Alhough the total pore volume remains unchanged (Table 1), a *t*-plot analysis revealed a significant decrease in the micropore volume. By comparing the N_2_ adsorption isotherm at 77 K and CO_2_ adsorption isotherm at 298 K, it becomes clear that the supermicropores have undergone an irreversible expansion after high pressure CO_2_ adsorption.

This is also confirmed by the pore size distribution analysis (Figure 10b) which indicates a decrease in the fraction of micropores and an increase in the content of mesopores. The higher contribution of the mesopores to the total surface area (Table 1) after high pressure CO_2_ adsorption (37.4% compared to 5.5% for the as-prepared carbon tubes (**4**)) from the *t*-plot analysis is consistent with results obtained from the pore size distribution analysis. The internal stress induced by the adsorbed CO_2_ under high pressure [49] might be one reason for the observed changes in the pore size distribution. Interestingly, changes in the pore structure induced by the high pressure CO_2_ adsorption are similar to the porosity changes caused by thermal annealing (Table 1). Annealing, similar to high pressure CO_2_ adsorption, leads to a decrease in the total micropore volume and an increase in the mesopore content.

**Figure 11:**
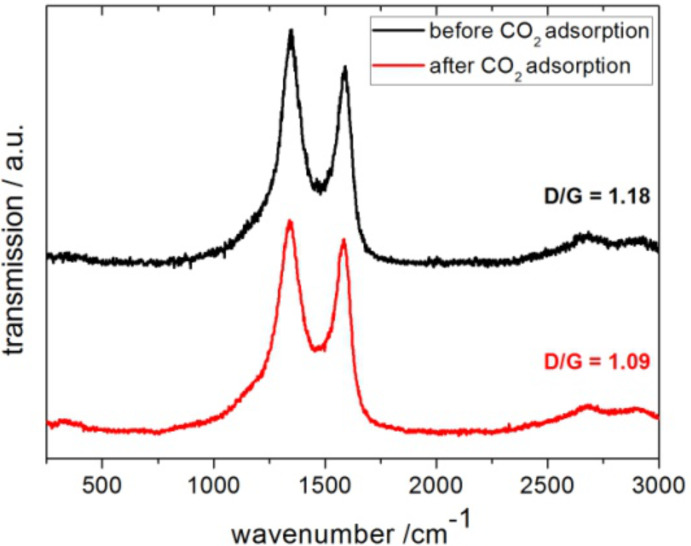
Raman spectra of carbon tubes (**4**) before (black/top) and after (red/bottom) high pressure CO_2_ adsorption.

The similarity of the carbon tube material (**4**) after CO_2_ adsorption with **4** carbonized at a high temperature of 1300 °C or 1600 °C is also observed in the Raman spectra (Figure 11). After the thermal annealing of **4**, the lowered D/G signal ratio indicates an increase in sp^2^ centers due to the growth of closed graphitic structures (onion-like or fullerene-type structures). SEM measurements have indicated that the tube morphology is still intact and unchanged after the CO_2_ adsorption cycling (not shown).

## Conclusion

One-dimensional mesoporous carbon tubes and silicon carbide tubes with diameters in the micrometer size range were synthesized using electrospun PS fibres (acting as both the carbon source as well as the template) and amorphous silica particles of spherical morphology derived from the Stoeber process as inorganic components. Due to the micro- and mesoporosity of the carbon tube walls and the macroporous inner space of the carbon tubes combined with the high specific surface area, this material is rendered highly suitable for high pressure carbon dioxide adsorption. However, high pressure CO_2_ adsorption was found to irreversibly change the pore structure of the carbon tubes, resulting in a decrease of the total fraction of supermicropores accompanied by an increase in the mesoporous content. Currently, work is underway to adjust the pore size and functionalize the carbon surface with different functional groups to further enhance and adjust the adsorption capacity.
